# The Effects of Risk Behaviors and Orthorexic Behavior on Glycemic Control in Adolescents with Type 1 Diabetes

**DOI:** 10.4274/jcrpe.galenos.2019.2019.0128

**Published:** 2020-09-02

**Authors:** Demet Taş, Eda Mengen, Pınar Kocaay, Seyit Ahmet Uçaktürk

**Affiliations:** 1 Ankara City Hospital, Children’s Hospital, Clinic of Pediatrics, Ankara, Turkey; 2 Ankara City Hospital, Children’s Hospital, Department of Pediatric Endocrinology, Ankara, Turkey

**Keywords:** Adolescent, risk behaviors, orthorexic eating behavior, glycemic control, type 1 diabetes

## Abstract

**Objective:**

Adolescents with chronic disease are as likely to exhibit risk-taking behavior as their peers. The aim was to investigate the risk behaviors of adolescents with type 1 diabetes (T1D) and the effect of orthorexic eating behaviors (OEB) on glycemic control (GC).

**Methods:**

This cross-sectional study was conducted with 107 adolescents with T1D, aged between 13-18 years and attending high school. The Risk Behavior Scale (RBS) and Orthorexic Behavior Scale (ORTO-11) were administered. A high RBS score indicates risky behavior; a low ORTO-11 score suggests a tendency to OEB. Participants hemoglobin A1c (HbA1c) status was used to assess GC: optimal GC (HbA1c ≤7%); or poor GC (HbA1c >7%).

**Results:**

Among females, those with poor GC had significantly lower (p=0.031) ORTO-11 scores than those with optimal GC, which was not the case in males. A significant correlation (r=0.358, p<0.001) was found between HbA1c and total RBS, eating habits subscale, and suicidal tendency subscale scores. Participants with poor GC had significantly higher eating habits subscale, alcohol use, and tobacco use subscale scores (p<0.05). Among females, total RBS and suicidal tendency subscale score was found to be significantly higher in those with poor GC; among males, alcohol subscale score was found to be significantly higher in those with poor GC.

**Conclusion:**

This study is the first to show the effect of the tendency for OEB on GC among female adolescents with T1D. The study showed that, along with inappropriate eating behaviors, adolescents with T1D should also be assessed for other risk behaviors to help achieve optimal GC.

**What is already known on this topic?**

Adolescents with chronic disease are as likely to exhibit risk-taking behavior as their peers. The risky behavior and disordered eating behavior of adolescents with type 1 diabetes (T1D), the most common chronic disease of adolescence, may result in serious negative consequences.

**What this study adds?**

This study investigated the effect of orthorexic eating behavior of adolescents with T1D on glycemic control (GC) for the first time. Assessing risk-taking behavior by gender, investigating the use of alcohol and tobacco by male adolescents with poor GC, and evaluating girls’ mood at each visit to the outpatient clinic, have been suggested as beneficial.

## Introduction

Adolescence is a period of rapid physical growth, sexual development, and psychosocial maturation. In the mid-adolescence period, which covers 13 to 17 years of age, peer norms take precedence over family norms, and the individual seeks peer-approval. In this period, an increase in impulsive behaviors is observed; feeling invincible, some adolescents may get involved in risky behaviors that may harm them. Throughout this process, change and maturation occurs in the adolescent brain. Compared with other age groups, adolescents are more susceptible to reward because of this developmental process of the brain. They may start smoking or using substances or engage in unsafe sexual behavior or unhealthy eating behaviors (1,2).

A 2010 study from the United States reported that 29.8% of high school students consumed alcohol, and 8.8% smoked. It was also found in the same study that 39.5% of adolescents were sexually active (3). A similar study from Asia in 2015 found the rate of smoking and alcohol consumption to be 22.3% and 27.9% (4), which were 32.3% and 18.7% in Turkey, respectively (5). The rate of suicide attempts was higher in adolescents than in other age groups (6). The higher rates may be due to psychological problems in adolescents such as anxiety and depression, stressful life (problems with peers or family, chronic illness, etc.) and personal characteristics (impulsivity, inadequate flexibility or resilience, etc.) (6). Adolescents often do not consider how they will be affected by risk behaviors, but this period usually passes without problems during healthy development. However, risk behaviors of adolescents with chronic diseases may have serious negative consequences (7). Adolescents exhibiting risk-taking behaviors were found to be less compliant to treatments (8). Type 1 diabetes (T1D) is the most common chronic disease of adolescence. In children with T1D, glycemic control (GC) is often found to be impaired during adolescence. Hormonal changes and physical growth during this period complicate metabolic control. Some studies emphasized the adverse effects of risk behaviors on GC in adolescents with T1D (9,10). Disordered eating behaviors (DEBs) are one of the risk behaviors in adolescents. DEBs include various behaviors to control body weight such as skipping meals, being a choosy-eater, vomiting after overeating, and improper exercise (11). DEBs may be asymptomatic in healthy adolescents; however, they may cause complications requiring immediate medical attention in adolescents with T1D. Previous studies found that adolescents with T1D have a higher rate of DEBs compared to their healthy peers and that the former group also had impaired GC (12,13). Orthorexia nervosa (ON) is defined as obsessive healthy-eating behavior and has not yet been defined or classified as an eating disorder (14). However, orthorexic eating behavior (OEB), tendency to orthoxic nervosa, might be risky for adolescents with T1D and negatively affect their GC. OEB has been previously investigated in healthy young people (15). The effect of OEB on GC has not been studied in adolescents with T1D. This study aimed to investigate the effect of risk behaviors and orthorexic tendencies on GC in adolescents with T1D.

## Methods

The study was conducted with patients between the ages of 13-18 who came to the pediatric endocrinology outpatient clinic in October, November, and December 2018. The Ethics Review Board of the University of Health Sciences Child Health and Diseases Hematology - Oncology Training and Research Hospital approved the study protocol (protocol number: 2018-130). The study was carried out in accordance with the Helsinki Declaration. Written consent for participation was obtained from adolescents and their parents. Adolescents over the age of 13 years, who had been followed up with a diagnosis of T1D for at least six months, using insulin, and attending high school were invited to the study. Adolescents from middle-income families (at least one parent had a permanent job, adolescent had room and phone, and expressed that they can get the recommended food) who live with both biological parents were included in the study. Those from low-income (parents had no work or received social assistance) and high-income (children attended a private school or had more than one car) families were excluded from the study. Adolescents who were underweight or obese, had another chronic disease, or psychiatric illness, were on medication other than insulin for any reason, or were hospitalized in the last three months, were also excluded from the study.

A total of 119 patients who met the inclusion criteria for the study were invited to participate; 107 patients agreed to participate in the study. Twelve adolescents did not consent to participate in the study. Two of the participants did not complete the scales; therefore, the demographic characteristics were analyzed based on 107 participants, and the data derived from scales were evaluated for 105 participants. Participants had a routine physical examination, necessary laboratory tests, and treatments similar to other patients with T1D. Weight was measured in kilograms (kg) using an electronic scale (Scale-Seca 220, Hamburg, Germany), height was measured in centimeters (cm) using the Harpenden stadiometer (Seritex, East Rutherford, NJ, USA). The standard deviations (SD) of height, weight, and body mass index (kg/m^2^) were calculated (16). Participants’ GCs were based on their hemoglobin A1c (HbA1c) level. At the time of blood sample collection for HbA1c, the Risk Behavior Scale (RBS) and Orthorexic Behavior Scale (ORTO-11) scale were administered to the participants. All participants completed these scales. They were instructed to fill out the questionnaires and put the forms in an envelope. Envelopes were deposited in a sealed box that was opened at the time of data analysis to provide anonymity. There was no time limit given to complete the scales. The relationships between whether patients had optimal GC (≤7%, ≤53 mmol/mol) or poor GC (>7%, >53 mmol/mol) and the patients’ age, age at diagnosis, and duration of disease were evaluated. The optimal-GC and poor-GC groups were also analyzed concerning their RBS and ORTO-11 scores.

### Glycemic Control

Glycated hemoglobin (HbA1c) measures a patient’s GC. HbA1c reflects the average serum glucose level in the period of the previous 3-4 months. HbA1c levels were analyzed by turbidimetric inhibition immunoassay using commercial kits (Beckman Coulter HbA1c) on clinical chemistry analyzer (Beckman Coulter AU 680, Brea, CA, USA). Clinical guidelines recommend an HbA1c level of no more than 7% (53 mmol/mol) for optimal GC (17,18). Based on these data, HbA1c values of ≤7% (53 mmol/mol) were taken to be the optimal GC, and values >7% (53 mmol/mol) were considered poor GC.

### Risk Behaviors Scale

The RBS has been developed by Gençtanırım and Ergene (19) to evaluate the risk behaviors of high-school students in Turkey. The RBS is a self-report scale with 36 five-point Likert items (5 = strongly agree, 4 = agree, 3 = partially agree, 2 = disagree, 1 = strongly disagree) where total score ranges from 36 to 180. Higher total scores indicate more risk behaviors. The RBS consists of six subscales: anti-social behaviors (I get involved in physical fighting, I get involved in bullying, I am teasing my friends, etc.), alcohol use (I drink alcohol when I want, I relax when I use alcohol, I drink alcohol to feel good, etc.), tobacco use (I smoke, I can get smokes if I want, my best friend smokes, etc.), suicidal tendency (I feel helpless in the face of problems, I feel pessimistic, I have low self-confidence, I wake up unhappy in the morning), eating habits (I like to eat junk food, I drink soft drinks every day, I eat mostly fast food, etc.), and school dropout (I think about dropping out of or taking a break from school, being successful in school does not help me, I want to work instead of going to school, etc.). High scores in each subscale indicate increased risk behaviors in a related area. Exploratory factor analysis of the RBS found item factor loads between 0.49 and 0.83. For reliability, the internal consistency coefficient (Cronbach alpha) was 0.91 for the whole scale and varied between 0.70 and 0.87 for the subscales.

### Orthorexia Nervosa Scale

The ORTO-15 scale was created by Donini et al (20) and was adapted to Turkish as the ORTO-11 by Arusoğlu et al (21). ORTO-11 is a Likert-type scale that includes 11 items (items 3, 4, 5, 6, 7, 8, 10, 11, 12, 13, and 14) from ORTO-15. The participants were asked to respond to ORTO-11 and to indicate how often the expression in each item identified themselves (always = 1, often = 2, sometimes = 3, never = 4). The total ORTO-11 score ranges from 11 to 44; a lower score indicates a tendency for OEB. The psychometric properties of ORTO-15 were investigated by factor analysis during its adaptation to Turkish; internal consistency coefficient was calculated to reveal factor loads varying between -0.44 and 0.69 for various items. Only the items with factor loads ≥0.50 were included in the Turkish version to generate an 11-item ORTO-11. The Cronbach’s alpha calculated for 15 items was 0.44 while it was 0.62 for the 11 items included in the Turkish language version.

### Statistical Analysis

The data were analyzed using SPSS software, version 23.0 for Windows (IBM Corp., Armonk, NY, USA). Descriptive statistics were presented as mean±SD, frequency, and percentage. Chi-square test was used to analyze categorical variables. The normal distribution of variables was tested using visual (histogram and probability graphs) and analytical methods (Kolmogorov-Smirnov or Shapiro-Wilk tests). Equality of variance was checked with the Levene test. Student’s t-test was used to compare the groups, and Pearson correlation coefficient was used to analyze the relationship between the variables when the parametric test prerequisites were met; Mann-Whitney U test and Spearman correlation coefficient were used otherwise. For all tests, the significance level was set at p<0.05.

## Results

Of the 107 adolescents diagnosed with T1D, 46.7% (n=50) were female, and 53.3% (n=57) were male. Other demographic data are presented in Table 1.

There was no significant difference between total RBS scores of males and females (p>0.05). Nonetheless, the alcohol use subscale score was significantly higher for males (p=0.003). On the contrary, the eating habits subscale scores were significantly higher in females (p=0.029). There was no significant difference between males and females regarding other RBS subscale scores (p>0.05). Total ORTO-11 score was significantly lower in females (t=-2.255, p=0.026).

The percentage of those who had a positive response to the item exploring smoking habits (strongly agree or agree) was 8.5% (n=9), of which 33.3% (n=3) were female and 66.7% (n=6) were male. The percentage of those who had a positive response to the item exploring alcohol use (strongly agree or agree) was 4.7% (n=5), all of whom were males.

A significant correlation was found between HbA1c and total RBS, eating habits subscale, and suicidal tendency subscale scores. No significant correlation was found between HbA1c and ORTO-11 score (p>0.05) (Table 2). There was also no significant correlation between ORTO-11 score and total RBS score or RBS subscales scores (p>0.05).

Of the participants, 18.7% (n=20) had an HbA1c of ≤7%; 81.3% (n=87) had an HbA1c of >7%. A significant relationship was found between HbA1c and patients’ age, patients’ age at diagnosis, and duration of disease (Table 3).

Those with an HbA1c of >7% had significantly higher eating habits subscale, alcohol use, and tobacco use subscale scores (p<0.05). There was no significant difference between those with high or low HbA1c regarding total RBS and other subscale scores (Table 4).

No significant difference was found between those with an HbA1c of >7% or ≤7% regarding gender distribution (χ^2^=1.920, p>0.05).

Among female participants, those with an HbA1c of >7% had significantly higher total RBS, suicidal tendency subscale, and eating habits subscale scores (p<0.05). Among females, the ORTO-11 score was significantly lower for those with an HbA1c of >7% (p<0.05). The ROC curve was calculated and used to determine whether the ORTO-11 score was a usable variable in determining the HbA1c value above 7% in the girls in our sample group. ROC analysis showed the area under the curve was low (AUC=0.54), the likelihood ratio (LR) + value (LR + value=1.8) was “weak” and the sensitivity of the test was low (20%).

Among male participants, those with an HbA1c of >7% had significantly higher eating habits and alcohol use subscale scores in RBS (p<0.05) (Table 5).

## Discussion

This study investigated, for the first time, the relationship between GC and the tendency for OEB among adolescents with T1D. Poor GC was shown to be associated with a tendency for OEB among female adolescents with T1D. In addition, this study indicated that adolescents’ risk behaviors and HbA1c were correlated. The suicidal tendency was higher among females with poor GC while alcohol use and tobacco use as risky behaviours were higher in males. As expected, unhealthy eating habits were found to be more common in patients with poor GC in both genders.

Results indicated that healthy eating habits should be emphasized for adolescents with T1D at each follow-up meeting. Unfortunately, there is a tendency to eat fewer fruits and vegetables and to eat unhealthy foods in all adolescents (22,23). Studies have shown that adolescents with T1D have similar eating behaviors to their peers (eating sweets, drinking carbonated drinks, and eating junk food) (24,25). Similar to this study, a review study recounted that adolescents with T1D often did not comply with their diets, and their GC was adversely affected (26). Clinical guidelines emphasize that adolescents with T1D should comply with their diet in order to have optimal metabolic control (17,18).

ON, or OEB, is defined as an obsession with healthy eating at a pathological level and has not yet met the criteria for an eating disorder. ON was first described by Bratman (27). Unlike other eating disorders, weight control is not the primary aim in OEB; however, over-occupation with healthy-eating practices is at a level that may impair one’s health (28). Similar to the other DEBs, a tendency for OEB was significantly higher among females than males (29). Portion control and calorie restriction, which are often advised for adolescents, might have increased female adolescents’ attention to nutritional issues. A previous study with 48 adolescents and young adults (aged 7-19 years) with T1D in Turkey indicated an increased tendency for OEB (30). We found that, among female adolescents with T1D, GC was found to be more impaired in those with a tendency for OEB; therefore, one cannot claim that they have healthier eating habits. A review study reported that ON was usually accompanied by psychopathology (31). In our study, no relationship was found between ORTO-11 scores and a suicidal tendency among female adolescents. ROC curve analysis was used to determine whether the ORTO-11 score was a usable variable in predicting poor GC in the girls in our sample group. As a result of this analysis, the area under the ROC curve was low, the LR + value was “weak” and the sensitivity of our test was low. For these reasons, it is concluded that ORTO-11 score is not a usable variable in determining the likelihood of an HbA1c value above 7. However, in our study, girls with poor GC tend to be more prone to OEB, suggesting that further investigation of this issue would be beneficial.

Among female adolescents with T1D, those with poor GC were found to have more suicidal tendencies. The suicidal tendency subscale of RBS includes the statements “I feel helpless in the face of problems”, “I feel pessimistic”, “I have low self-confidence”, and “I wake up unhappy in the morning”, which reflect depressive feelings and thoughts. Depressive mood and low self-esteem were associated with suicidal ideation. In previous studies, it was also reported that depressive symptoms in adolescents with T1D had negatively affected their GC (32,33). The results of our study were consistent with those findings. Studies have indicated that negative mood is more common in female adolescents with T1D compared to their male counterparts (34,35,36). However, contrary to these studies, no significant difference was found between the suicidal tendency subscale scores of male and female adolescents in our study.

When males and female were evaluated separately, the suicidal tendency of females with poor GC was higher than those with optimal GC, whereas such a difference was not observed in males. Similarly, it has previously been reported that, among adolescents with T1D who had negative mood, females had a worse GC than males (35). In the pediatric endocrinology department where this study was conducted, psychiatric consultation is requested for every patient with poorly controlled diabetes. The significant association between poor GC and suicidal tendency that was identified, despite the exclusion of patients with any psychiatric diagnosis, is remarkable. This finding may suggest that the resilience in the face of chronic disease and compliance to treatment were worse among our female patients.

Similar to previous studies (10,37,38), it was found that alcohol consumption and smoking in adolescents diagnosed with T1D had a negative effect on GC in our sample. In contrast, a previous Turkish study found no relationship between GC and alcohol consumption or smoking in another study with adolescents with T1D (39). In our study, the rates of smoking and alcohol consumption rates were much lower than the rates in the studies that found a relationship (10,37,38). The relationship between smoking and alcohol use and serious health outcomes of adolescents diagnosed with T1D is well-known. Alcohol consumption of adolescents with T1DM increases the risk of hospitalization due to ketoacidosis. Even in small amounts, it can increase the risk of hypoglycemia within 1-2 days (11,40,41). Smoking in adolescents with T1DM has been shown to impair blood sugar regulation. In addition, tobacco use will aggravate diabetes and tobacco-related complications over time (42).

The lack of alcohol use among female adolescents in our study was probably related to cultural factors. In Turkey, the rate of alcohol consumption is much lower among healthy female adolescents than their male counterparts (43). The age range of adolescents with poor GC in our study corresponds to the beginning of middle adolescence and high school. The process of adaptation to new friends and social environment may have influenced their eating behavior and GC. Their compliance with treatment may improve after 1-2 years of getting used to their new school and peers. Perhaps due to the developmental characteristics of adolescence, our rate of impaired GC patients was high (81.3% of the cohort). Sociocultural factors other than school life, like the influence of family and peer relationships, may also have affected their behavior. Adolescents diagnosed with T1D at a younger age and had a long duration of disease were found to have worse GC. Metabolic control interventions should be performed differently depending on age, duration of disease and age of onset of disease.

### Study Limitations

Our study has several limitations. Improper sexual behavior is an important risk behavior, and the lack of data on the sexual behavior of adolescents in our study was a significant limitation. One of the risks that affect GC is the inappropriate injection of insulin; lack of data on adolescents’ compliance with insulin treatment was also a significant limitation. A review study indicated that OEN was accompanied by various obsessive disorders (31). Although adolescents with existing psychiatric diagnoses were not included in the study, we did not investigate the presence of obsessive disorders and other psychopathologies in our study.

## Conclusion

In conclusion, this study has demonstrated the importance of considering the relationship between GC and risk behaviors in adolescents with T1D. Higher incidence of OEB in female participants with poor GC suggests that adolescents with T1D should be evaluated for nutritional habits, including an obsession with healthy eating. Evaluating nutritional habits according to their development, assessing depressive symptoms repeatedly, and asking about alcohol and tobacco use based on gender may contribute to disease management in adolescents with T1D who have poor GC. The fact that bad eating habits are one of the most critical problems of adolescents with T1D necessitates the further investigation of this issue. Future studies should address the incidence of OEB and the psychopathologies that may accompany OEB and influence GC in adolescents with T1D.

## Figures and Tables

**Table 1:**
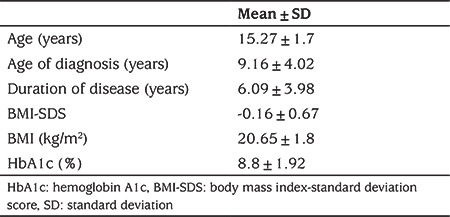
Demographic data of participants

**Table 2:**
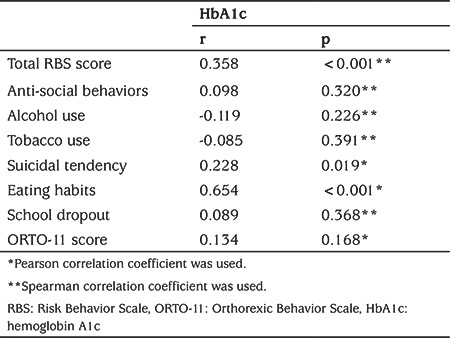
Correlation analysis between Risk Behavior Scale and Orthorexic Behavior Scale-11 scale scores and hemoglobin A1c

**Table 3:**
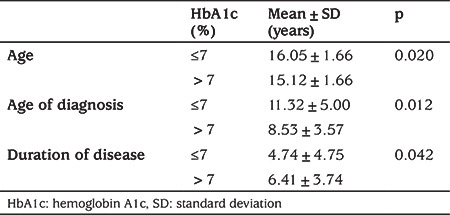
Age, age of diagnosis and duration of disease of participants according to hemoglobin A1c level

**Table 4:**
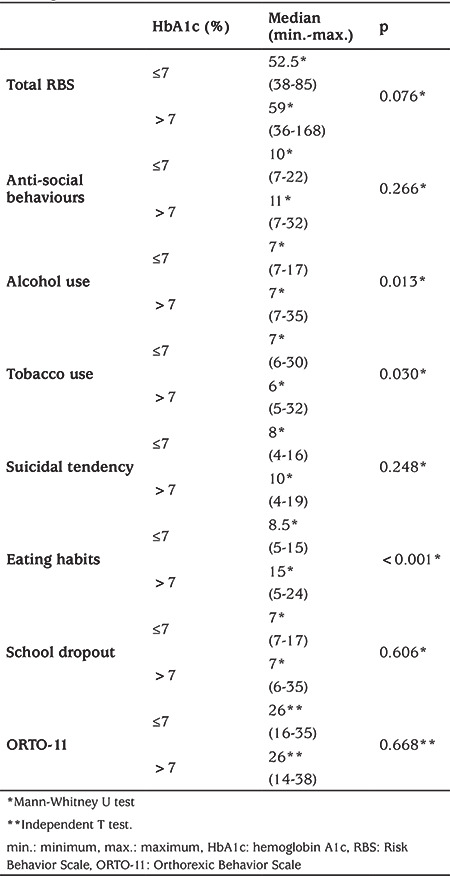
Risk Behavior Scale total and subscale scores and Orthorexic Behavior Scale-11 scale score according to hemoglobin A1c level

**Table 5:**
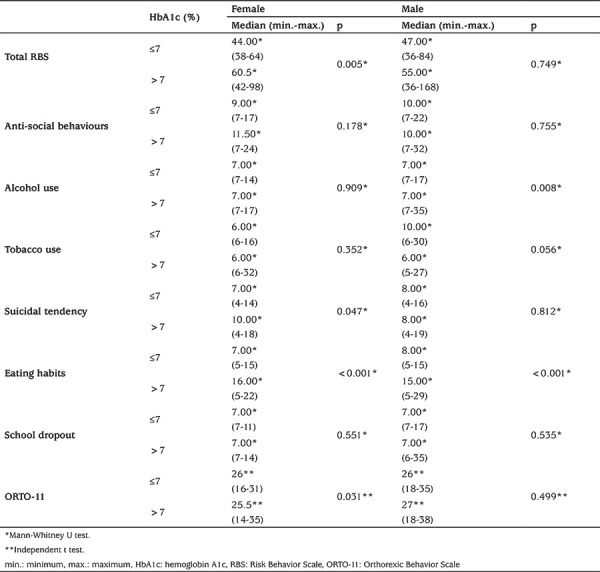
Risk Behavior Scale total and subscale scores and Orthorexic Behavior Scale-11 score according to gender, related to hemoglobin A1c level

## References

[1] Romer D (2010). Adolescent risk taking, impulsivity, and brain development: Implications for prevention. Dev Psychobiol.

[2] Neinstein LS (2008.). Adolescent health care: a practical guide. Lippincott Williams & Wilkins.

[3] E Eaton DK, Kann L, Kinchen S, Shanklin S, Ross J, Hawkins J, Harris WA, Lowry R, McManus T, Chyen D, Lim C, Whittle L, Brener ND, Wechsler H;, Centers for Disease Control and Prevention (CDC) (2010). Youth risk behavior surveillance-United States. 2009. MMWR Surveill Summ.

[4] Sirirassamee T, Sirirassamee B (2015). Health risk behavior among Thai youth: national survey 2013. Asia Pac J Public Health.

[5] Terzi O, Dundar C (2016). Tobacco, Alcohol, Substance use Patterns and Associated Risk Factors in a Representative Sample of High School Students in Corum, Turkey. Austin J Public Health Epidemiol.

[6] Carballo JJ, Llorente C, Kehrmann L, Flamarique I, Zuddas A, Purper- Ouakil D, Hoekstra PJ, Coghill D, Schulze UME, Dittmann RW, Buitelaar JK, Castro-Fornieles J, Lievesley K, Santosh P, Arango C;, STOP Consortium (2020). Psychosocial risk factors for suicidality in children and adolescents. Eur Child Adolesc Psychiatr.

[7] Louis-Jacques J, Samples C (2011). Caring for teens with chronic illness: risky business?. Curr Opin Pediatr.

[8] Rapoff MA (2010). Consequences of Nonadherence and Correlates of Adherence. In: Adherence to Pediatric Medical Regimens. Issues in Clinical Child Psychology. Springer, Boston, MA,.

[9] Frey MA, Guthrie B, Loveland-Cherry C, Park PS, Foster CM (1997). Risky behavior and risk in adolescents with IDDM. J Adolesc Health.

[10] Hogendorf AM, Fendler W, Sierosławski J, Bobeff K, Węgrewicz K, Malewska KI, Przudzik MW, Szmigiero-Kawko M, Sztangierska B, Myśliwiec M, Szadkowska A, Młynarski WM (2017). Alcohol and cigarette use among adolescents with type 1 diabetes. Eur J Pediatr.

[11] Rosendahl J, Bormann B, Aschenbrenner K, Aschenbrenner F, Strauss B (2009). Dieting and disordered eating in German high school athletes and non‐athletes. Scand J Med Sci Sports.

[12] Hanlan ME, Griffith J, Patel N, Jaser SS (2013.). Eating disorders and disordered eating in type 1 diabetes: prevalence, screening, and treatment options. Curr Diab Rep.

[13] Pinar R (2005). Disordered eating behaviors among Turkish adolescents with and without Type 1 diabetes. J Pediatr Nurs.

[14] Fugh-Berman A (2001). Health food junkies: Orthorexia Nervosa: Overcoming the obsession with healthful eating. JAMA.

[15] Hyrnik J, Janas-Kozik M, Stochel M, Jelonek I, Siwiec A, Krysta K, Rybakowski JK (2016). Prevalence of orthorexia nervosa among polish adolescents–Assessment made by the ORTO-15 Questionnaire. European Psychiatry.

[16] Neyzi O, Bundak R, Gökçay G, Günöz H, Furman A, Darendeliler F, Baş F (2015). Reference values for weight, height, head circumference, and body mass index in Turkish children. J Clin Res Pediatr Endocrinol.

[17] DiMeglio LA, Acerini CL, Codner E, Craig ME, Hofer SE, Pillay K, Maahs DM (2018). ISPAD Clinical Practice Consensus Guidelines 2018: Glycemic control targets and glucose monitoring for children, adolescents, and young adults with diabetes. Pediatr Diabetes.

[18] No authors listed (2019). Children and Adolescents: Standards of Medical Care in Diabetes-2019. Diabetes Care.

[19] Gençtanırım D, Ergene T (2014). Riskli davranışlar ölçeğinin geliştirilmesi: Geçerlik ve güvenirlik çalışmaları. Int J Human Soc.

[20] Donini LM, Marsili D, Graziani MP, Imbriale M, Cannella C (2005). Orthorexia nervosa: validation of a diagnosis questionnaire. Eat Weight Disord.

[21] Arusoğlu G, Kabakçi E, Köksal G, Merdol TK (2008). Orthorexia Nervosa and Adaptation of ORTO-11 into Turkish. Turk Psikiyatri Derg.

[22] Story M, Neumark-Sztainer D, French S (2002). Individual and environmental influences on adolescent eating behaviors. J Am Diet Assoc.

[23] Demirezen E, Coşansu GJSTED (2005). Adölesan çağı öğrencilerde beslenme alışkanlıklarının değerlendirilmesi. Sürekli Tıp Eğitimi Dergisi.

[24] Husárová D, Kostičová M, Kočišová D, Schusterová I, Gecková AM (2017). Do Adolescents with T1DM Differ from Their Peers in Health, Eating Habits and Social Support?. Cent Eur J Public Health.

[25] Øverby NC, Margeirsdottir HD, Brunborg C, Dahl-Jørgensen K, Andersen LF;, Norwegian Study Group for Childhood Diabetes (2008). Sweets, snacking habits, and skipping meals in children and adolescents on intensive insulin treatment. Pediatr Diabetes.

[26] Patton SR (2011). Adherence to diet in youth with type 1 diabetes. J Am Diet Assoc.

[27] Bratman S. Original essay on orthorexia. 1997. Retrieved March 9, 2015. Available from:.

[28] Brytek-Matera A (2012). Orthorexia nervosa–an eating disorder, obsessive-compulsive disorder or disturbed eating habit. Arch Psychiatr Psychotherapy.

[29] Takii M, Uchigata Y, Kishimoto J, Morita C, Hata T, Nozaki T, Kawai K, Iwamoto Y, Sudo N, Kubo C (2011). The relationship between the age of onset of type 1 diabetes and the subsequent development of a severe eating disorder by female patients. Pediatr Diabetes.

[30] Fidan T, Orbak Z, Karabağ K, Koçak K (2017). Orthorexia Nervosa and Family Functionality in Children and Adolescents with Type 1 Diabetes Mellitusu olan Çocuk ve Ergenlerde Ortoreksiya Nervosa ve Aile İşlevselliği. Osmangazi Tıp Dergisi.

[31] McComb SE, Mills JS (2019). Orthorexia nervosa: A review of psychosocial risk factors. Appetite.

[32] Santos FRM, Bernardo V, Gabbay MA, Dib SA, Sigulem D (2013). The impact of knowledge about diabetes, resilience and depression on glycemic control: a cross-sectional study among adolescents and young adults with type 1 diabetes. Diabetol Metab Syndr.

[33] Hood KK, Rausch JR, Dolan LM (2011). Depressive symptoms predict change in glycemic control in adolescents with type 1 diabetes: rates, magnitude, and moderators of change. Pediatr Diabetes.

[34] Glick BA, Hong KMC, Obrynba K, Kamboj MK, Hoffman RP (2018). Identifying depressive symptoms among diabetes type and the impact on hemoglobin A1c. J Pediatr Endocrinol Metab.

[35] La Greca AM, Swales T, Klemp S, Madigan S, Skyler J (1995). Adolescents with diabetes: Gender differences in psychosocial functioning and glycemic control. Child Health Care.

[36] Hood KK, Rausch JR, Dolan LM (2011). Depressive symptoms predict change in glycemic control in adolescents with type 1 diabetes: rates, magnitude, and moderators of change. Pediatr Diabetes.

[37] Snyder LL, Truong YKN, Law JR (2016). Evaluating substance use and insulin misuse in adolescents with type 1 diabetes. Diabetes Educ.

[38] Scaramuzza A, De Palma A, Mameli C, Spiri D, Santoro L, Zuccot GV (2010). Adolescents with type 1 diabetes and risky behaviour. Acta Paediatr.

[39] Yetim A, Alikaşifoğlu M, Baş F, Eliaçık K, Çığ G, Erginöz E, Ercan O, Bundak R (2018). Glycemic control and health behaviors in adolescents with type 1 diabetes. Turk J Pediatr.

[40] Hermann JM, Meusers M, Bachran R, Kuhnle-Krahl U, Jorch N, Hofer SE, Holl RW;, DPV initiative (2017). Self-reported regular alcohol consumption in adolescents and emerging adults with type 1 diabetes: A neglected risk factor for diabetic ketoacidosis? Multicenter analysis of 29 630 patients from the DPV registry. Pediatr Diabetes.

[41] Jaser SS, Yates H, Dumser S, Whittemore R (2011). Risky business: Risk behaviors in adolescents with type 1 diabetes. Diabetes Educ.

[42] Hofer SE, Rosenbauer J, Grulich-Henn J, Naeke A, Fröhlich-Reiterer E, Holl RW; DPV-Wiss (2009). Study Group. Smoking and metabolic control in adolescents with type 1 diabetes. J Pediatr.

[43] Kara B, İşcan B (2016). Predictors of health behaviors in Turkish female nursing students. Asian Nurs Res (Korean Soc Nurs Sci).

